# The effect of phacoemulsification performed with vitrectomy on choroidal vascularity index in eyes with vitreomacular diseases

**DOI:** 10.1038/s41598-021-99440-4

**Published:** 2021-10-06

**Authors:** Heejeong Chun, Joo Young Kim, Jae Hyuck Kwak, Rae Young Kim, Mirinae Kim, Young-Gun Park, Young-Hoon Park

**Affiliations:** 1 Department of Ophthalmology and Visual Science, Seoul St. Mary’s Hospital, College of Medicine, The Catholic University of Korea, Seoul, South Korea; 2 Catholic Institute for Visual Science, The Catholic University of Korea, Seoul, South Korea

**Keywords:** Outcomes research, Retinal diseases, Eye diseases

## Abstract

This study evaluated the effects of cataract surgery combined with pars plana vitrectomy (ppV) on choroidal vascularity index (CVI) in eyes with epiretinal membrane (ERM) and full thickness macular hole (FTMH). Medical records of 132 eyes with ERM or FTMH were retrospectively reviewed and classified into a ppV group and a ppV combined with cataract surgery group (phaco + ppV group). The CVI were measured at baseline, 1, 3 and 6 months after the surgery, using the selected swept-source (SS) optical coherence tomography (OCT) scan passing through the central fovea, which was then segmented into luminal and stromal area by image binarization. The mean CVI of phaco + ppV group were 61.25 ± 1.97%, 61.66 ± 1.81%, and 62.30 ± 1.92% at baseline, 1 and 3 months, respectively (*p* < 0.001). The mean CVI of ppV group were 62.69 ± 1.92%, 62.03 ± 1.51%, and 61.45 ± 1.71% at baseline, 1 and 3 months, respectively (*p* < 0.001). The final CVI were measured at 6 months and compared with the baseline CVI. The mean CVI of phaco + ppV group were 61.21 ± 1.99% at baseline and 60.68 ± 2.02% at 6 months (*p* < 0.001). The mean CVI of ppV group were 62.93 ± 1.70% at baseline and 61.77 ± 1.74% at 6 months (*p* < 0.001). Vitrectomy significantly decreases CVI in vitreomacular diseases possibly due to the removal of vitreomacular traction or postoperative oxygenation change in the eye. On the contrary, combined surgery of vitrectomy and cataract surgery significantly increases CVI in the early stage of postoperative period, which suggests choroidal vascular dilatation or congestion due to postoperative inflammation. Although the CVI were measured lower than the baseline in the end, more thorough inflammation control may be essential after combined surgery.

## Introduction

Posterior vitreous detachment, a result from liquefaction and separation of the vitreous body from the internal limiting membrane of the retina, is a normal process with increasing age^1^. However, a vitreomacular adhesion can arise when the posterior vitreous separation is incomplete^2,3^, which may lead to vitreomacular interface diseases such as vitreomacular traction syndrome, macular hole, epiretinal membrane and more^4–6^. Epiretinal membrane (ERM) is an abnormal membrane which appears on the surface of the internal limiting membrane of the retina^7^. Full-thickness macular hole (FTMH) is a defect involving full thickness in the foveal area from internal limiting membrane (ILM) to the outer segment of photoreceptor layer^8^. The prevalence of these vitreomacular interface diseases has been rising over the years^9^.

Optical coherence tomography (OCT) has been a crucial tool since its introduction for diagnosis and follow-up of many vitreoretinal diseases by providing high-resolution cross-sectional images of the retinal layers^10–12^. As imaging techniques improved, OCT now enables deeper penetration of the retinal pigment epithelium (RPE) and better visualization of the choroidal–scleral interface using longer wavelengths^13,14^. This advancement in OCT provides an opportunity to evaluate choroidal changes more accurately in various vitreomacular diseases.

The choroid is composed of highly vascularized structure, which supplies the outer retina and the RPE with oxygen and nutrients^15^. Choroidal thickness (CT) has been reported under numerous studies for its alteration in various ocular and systemic diseases^16–19^. However, CT change alone does not explain which part of the choroid is being affected by the disease. Taking a step forward from evaluating the CT changes, recent studies are now investigating the choroidal vascularity index (CVI), which is the ratio of the vascular luminal area (LA) to the total choroidal area (TCA)^20–23^. The CVI thus can measure the choroidal vascular components quantitatively, which then can provide a more accurate assessment on changes in choroidal vascularity. Several studies have used the CVI to evaluate the effects of various ocular diseases with inflammatory reaction or blood flow disturbance such as uveitis or diabetic retinopathy on choroidal vascular structures^24–26^.

Even though alternative nonsurgical treatments have been explored under several studies, vitrectomy is still considered as the standard treatment for symptomatic vitreomacular adhesion in a practical setting^27,28^. Because of the rising prevalence of vitreomacular interface diseases, a need for vitrectomy has also been increasing, and the effect of vitrectomy on choroidal structures can be an intriguing study issue. Previous study has produced results showing that vitreomacular surgery significantly decreases CVI^29^. In addition, a significant increase of CVI has been noticed after cataract surgery using phacoemulsification^30,31^. However, in many cases, vitrectomy is performed with cataract surgery using phacoemulsification since it provides good visualization during the vitrectomy and avoids patients’ inconvenience of having a second surgery for cataract^32^. This has led us to a question how the choroidal vascular structure will change when phacoemulsification is performed at the same time as vitrectomy.

The purpose of our study is to evaluate the effects of cataract surgery using phacoemulsification combined with pars plana vitrectomy (ppV) on CVI in eyes with vitreomacular diseases such as ERM and FTMH.

## Results

This study included a total of 132 patients. Demographic and ophthalmic characteristics of the study participants are summarized in Table 1. The mean age of the patients was 65.5 ± 7.0 years, and 46 of the 132 patients were male (34.8%). 19 and 54 patients had diabetes and hypertension, respectively. The mean refractive errors were -0.47 ± 1.63 diopters and the mean BCVA were 0.57 ± 0.27 logMAR at initial visit. They had the mean intraocular pressure of 14.60 ± 2.97 mmHg, the mean axial lengths of 23.75 ± 1.12 mm, and the mean SFCT of 296.52 ± 63.10 μm.

**Table 1:** Demographic and clinical characteristics of the study participants at baseline.

	Total (n = 132)	ERM (n = 67)		FTMH (n = 65)	
		Phaco + ppV (n = 34)	ppV (n = 33)	Phaco + ppV (n = 30)	ppV (n = 35)
Age, years	65.50 ± 7.0	65.74 ± 6.52	68.36 ± 6.62	64.83 ± 6.36	63.14 ± 7.59
Gender, male	46	11	17	7	11
Diabetes	19	0	7	9	3
Hypertension	54	11	13	16	14
SE, diopter	− 0.47 ± 1.63	− 0.64 ± 2.14	− 0.59 ± 0.97	− 0.51 ± 2.09	− 0.44 ± 1.07
BCVA, logMAR	0.57 ± 0.27	0.49 ± 0.20	0.40 ± 0.10	0.73 ± 0.25	0.65 ± 0.29
IOP, mmHg	14.60 ± 2.97	10.91 ± 3.27	13.42 ± 2.19	15.20 ± 2.72	14.91 ± 3.32
AL, mm	23.75 ± 1.12	23.95 ± 1.13	23.65 ± 0.87	23.51 ± 1.03	23.85 ± 1.37
SFCT, μm	296.52 ± 63.10	315.50 ± 68.07	275.73 ± 57.75	300.30 ± 65.19	294.43 ± 57.31

Data are expressed as mean ± standard deviation (95% confidence interval).

Abbreviations: AL, axial length; BCVA, best-corrected visual acuity; ERM, epiretinal membrane; FTMH, full thickness macular hole; IOP, intraocular pressure; logMAR, logarithm of the minimum angle of resolution; Phaco, phacoemulsification; ppV, pars plana vitrectomy; SE, spherical equivalent; SFCT, subfoveal choroidal thickness.

In the ppV group, 39 pseudophakic eyes and 29 phakic eyes were included. The 29 phakic eyes in this group have not yet showed significant cataract progression during the 6 months follow-up, which in such case could affect the OCT image quality for accurately measuring CVI.

**Figure 1:**
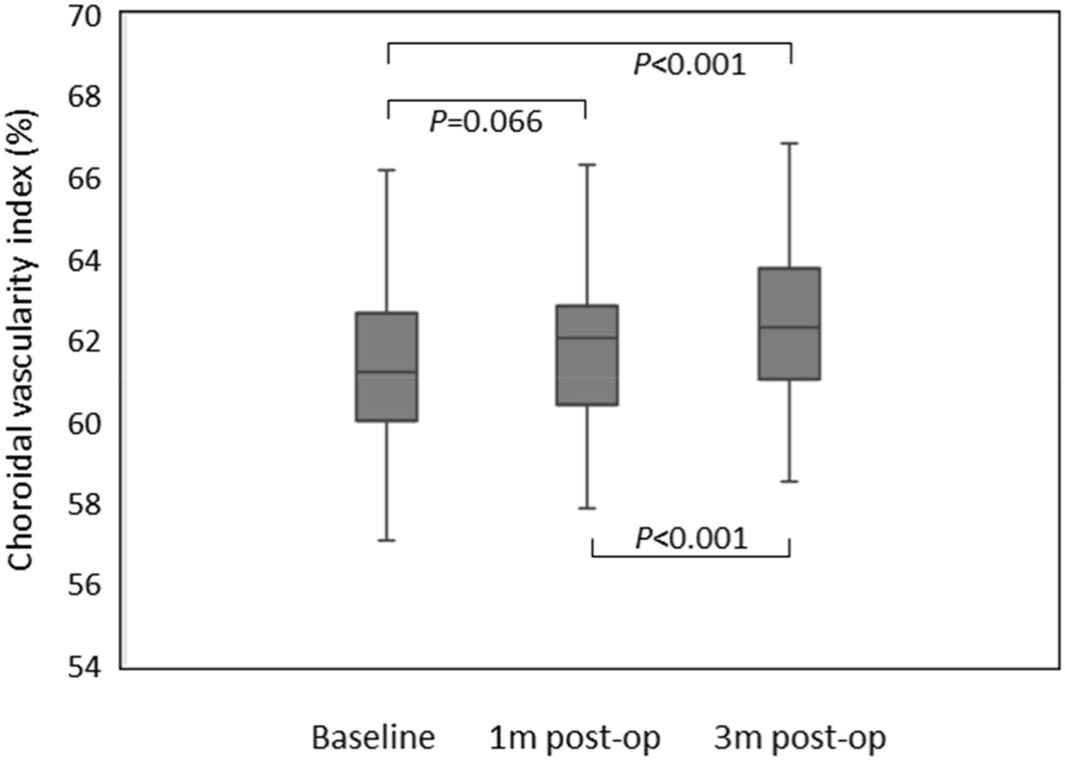
Choroidal vascularity index in Phaco + ppV group evaluated by repeated measures ANOVA tests and post hoc analysis. Increase in choroidal vascularity index was statistically significant from baseline to 3 months post-op and from 1 month post-op to 3 months post-op.

**Figure 2:**
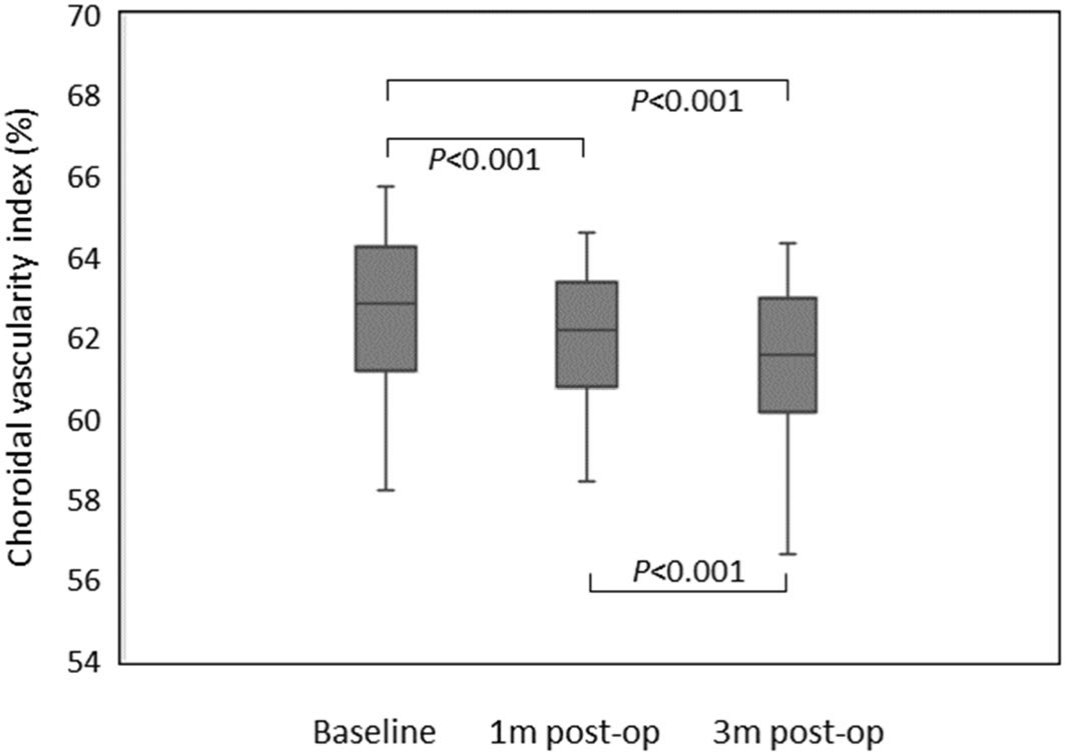
Choroidal vascularity index in ppV group evaluated by repeated measures ANOVA tests and post hoc analysis. Reduction in choroidal vascularity index was statistically significant between every group.

The mean CVI of phaco + ppV group were 61.25 ± 1.97%, 61.66 ± 1.81%, and 62.30 ± 1.92% at baseline, 1 and 3 months, respectively. In this phaco + ppV group, the ANOVA test showed *p* < 0.001, df = 2, and the p value of Tukey’s multiple comparisons test for baseline versus 1 month was 0.066, and for baseline versus 3 months and 1 month versus 3 months were < 0.001 (Fig. 1). The mean CVI of ppV group were 62.69 ± 1.92%, 62.03 ± 1.51%, and 61.45 ± 1.71% at baseline, 1 and 3 months, respectively. In this ppV group, the ANOVA test showed *p* < 0.001, df = 2, and the p value of Tukey’s multiple comparisons test for baseline versus 1 month, baseline versus 3 months, and 1 month versus 3 months were all < 0.001 (Fig. 2). We also repeated the above analysis after separating the study patients into ERM and FTMH groups. The ANOVA test result in ERM with phaco + ppV group was *p* = 0.006 and in ERM with ppV group was *p* = 0.002. The ANOVA test result in FTMH with phaco + ppV group was *p* < 0.001 and in FTMH with ppV group was *p* < 0.001. These results showed that the mean CVI were significantly increased after phaco + ppV and decreased after ppV (Table 2) until 3 months of postoperative period.

**Table 2:** Choroidal vascularity index (%) changes from baseline to 3 months post-op.

CVI	Phaco + ppV (n = 64)	ppV (n = 68)	Phaco + ppV (n = 64)		ppV (n = 68)	
			ERM (n = 34)	FTMH (n = 30)	ERM (n = 33)	FTMH (n = 35)
Baseline pre-op	61.25 ± 1.97	62.69 ± 1.92	61.80 ± 2.05	60.63 ± 1.69	62.66 ± 1.85	62.71 ± 2.01
1 m post-op	61.66 ± 1.81	62.03 ± 1.51	61.97 ± 1.94	61.31 ± 1.61	62.36 ± 1.53	61.73 ± 1.44
3 m post-op	62.30 ± 1.92	61.45 ± 1.71	62.59 ± 2.02	61.96 ± 1.78	62.16 ± 1.75	61.20 ± 1.61
*P*-value	** < 0.001**	** < 0.001**	**0.006**	** < 0.001**	**0.002**	** < 0.001**
Eta^2	0.213	0.283	0.153	0.298	0.199	0.386

Data are expressed as mean ± standard deviation (95% confidence interval). Repeated measures ANOVA tests and post hoc analysis. Statistically significant *P*-values are highlighted as bold.

Abbreviations: ERM, epiretinal membrane; FTMH, full thickness macular hole; Phaco, phacoemulsification; ppV, pars plana vitrectomy.

**Figure 3:**
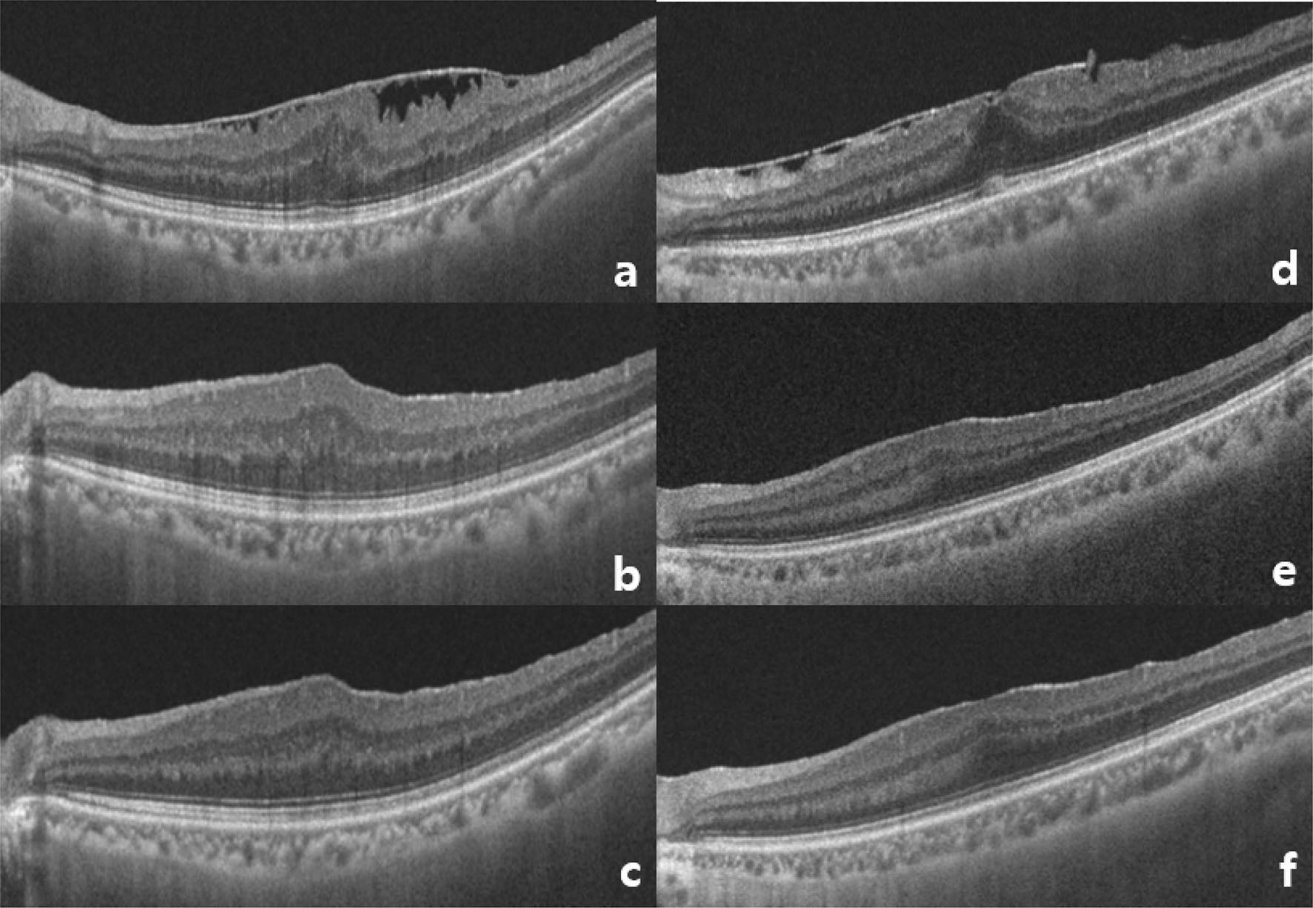
Optical coherence tomography (OCT) B-scans taken from patients with epiretinal membrane (ERM), one from a 77-year-old male who had phaco + ppV (a. baseline CVI = 60.92%, b. 3 months post-op CVI = 61.12%, c. 6 months post-op CVI = 59.40%) and another from a 56-year-old female who had ppV (a. baseline CVI = 61.08%, b. 3 months post-op CVI = 60.03%, c. 6 months post-op CVI = 60.01%). Choroidal vascularity index in phaco + ppV group increased until 3 months after surgery but dropped to below baseline at 6 months. Choroidal vascularity index in ppV group showed a constant decrease until 6 months after surgery.

We measured the final CVI at 6 months and compared with the baseline CVI, excluding 3 patients from phaco + ppV group and 4 patients from ppV group who did not revisit our clinic at 6 months of time. The mean CVI of phaco + ppV group were 61.21 ± 1.99% at baseline and 60.68 ± 2.02% at 6 months, with the paired t-test of *p* < 0.001. The mean CVI of ppV group were 62.93 ± 1.70% at baseline and 61.77 ± 1.74% at 6 months, with the paired t-test of *p* < 0.001 (Table 3). These results showed that the mean CVI were significantly decreased in both phaco + ppV and ppV groups at 6 months, even though phaco + ppV group showed increased CVI until 3 months after the surgery. OCT B-scans taken from patients with ERM who had phaco + ppV or ppV are presented with its CVI values (Fig. 3).

**Table 3:** Choroidal vascularity index (%) at 6 months post-op.

CVI	Phaco + ppV (n = 61)	ppV (n = 64)	Phaco + ppV (n = 61)		ppV (n = 64)	
			ERM (n = 31)	FTMH (n = 30)	ERM (n = 32)	FTMH (n = 32)
Baseline pre-op	61.21 ± 1.99	62.93 ± 1.70	61.77 ± 2.11	60.63 ± 1.69	62.79 ± 1.72	63.06 ± 1.69
6 m post-op	60.68 ± 2.02	61.77 ± 1.74	61.03 ± 2.10	60.32 ± 1.89	61.84 ± 1.91	61.70 ± 1.57
*P*-value	** < 0.001**	** < 0.001**	**0.001**	**0.027**	** < 0.001**	** < 0.001**

Data are expressed as mean ± standard deviation (95% confidence interval). Paired t-test analysis. Statistically significant *P*-values are highlighted as bold.

Abbreviations: ERM, epiretinal membrane; FTMH, full thickness macular hole; Phaco, phacoemulsification; ppV, pars plana vitrectomy.

Among the 132 study patients, 50 ERM and 51 FTMH patients with normal fellow eyes were selected and analyzed by comparing the CVI of the eyes with vitreomacular disease (ERM and FTMH) and the normal fellow eyes. The mean CVI of the eyes with ERM were 62.29 ± 0.26% and that of the normal fellow eyes were 62.47 ± 2.09%. The mean CVI of the eyes with FTMH were 61.67 ± 2.20% and that of the normal fellow eyes were 62.23 ± 2.67%. The t-test result in ERM versus normal eye group was *p* = 0.649, and in FTMH versus normal eye group was *p* = 0.253.

## Discussion

The choroidal structure can now be investigated more accurately by segmenting it into choroidal stroma and vascular components. In this study, we evaluated the effects of cataract surgery using phacoemulsification combined with vitrectomy on the vascular components of the choroid, represented as CVI, in eyes with vitreomacular diseases such as ERM and FTMH. Our results showed that from right after the surgery until 3 months, the CVI significantly decreased after vitrectomy but increased after vitrectomy combined with cataract surgery using phacoemulsification. However, the CVI eventually decreased in both surgery groups and at 6 months of postoperative period, the CVI in both groups were measured significantly lower than the baseline CVI.

In our study, eyes with ERM or FTMH did not show significant difference in CVI from the normal fellow eyes. This result did not agree with the previous study, which suggested that vitreomacular traction changed the choroidal vascularity structures through various mechanisms and showed a significantly different CVI between the affected eyes and the normal fellow eyes^29^. However, other studies^33,34^ have also proposed that normal fellow eyes of unilateral ERM showed similar tendency in retinal blood flow change as the affected eyes. It might be possible that the changes in choroid level caused from vitreomacular disease also could affect the choroid in the fellow eyes, which needs to be investigated further.

Even though the effect of ERM and FTMH on the CVI did not concur, Rizzo et al.^29^ has presented a decrease in CVI after vitrectomy for ERM and FTMH, in which our study results coincide. Rizzo et al.^29^ suggested that vitreomacular traction may cause low grade or inflammation in choroid level, and our study may suggest removal of this traction can subside this inflammation and result in the choroidal structure changes, especially its vascularity, regardless of the choroid’s preoperative state. Also, previous studies showed that vitrectomy reduces ocular blood flow in diabetic retinopathy patients^35^ and increases partial pressure of oxygen in posterior segment of the eye^36^. These study results could suggest that postoperative oxygenation change in the posterior segment of the eye could affect the choroidal vascular structures.

It has been known through many literatures that cataract surgery induces intraocular inflammation throughout anterior and posterior ocular segments, possibly resulting in many complications such as posterior capsular opacification or cystoid macular edema^37–39^. Several studies evaluated the effect of cataract surgery on the choroidal thickness and found out that the choroidal thickness mostly increased after cataract surgery^40–42^. However, their results did not agree on the period in which the increase of the choroidal thickness persisted. According to Noda et al.^40^, the choroidal thickness remained increased at least for 6 months, whereas Ibrahim et al.^41^ reported that the increased choroidal thickness returned to baseline after 3 months post cataract surgery. Recent studies have expanded the field by investigating the alterations in the vascular components of the choroid and showed that the CVI increased after cataract surgery^30,31^. Therefore, it could be speculated that the choroidal structure change after cataract surgery is mainly the change of its vascular components, due to the intraocular inflammation caused by the surgical trauma. According to previous studies, the choroidal vascular components could also be increased by various inflammatory ocular diseases, such as HLA-B27-associated uveitis or panuveitis^24,25^.

Another possible cause of postoperative CVI changes might be related to the IOP control during and after the surgery. Previous study^30^ has suggested that the increased CVI after cataract surgery could be caused by the IOP decrease after the surgery. The reduced IOP leads to increased ocular perfusion pressure, which causes CVI to increase. Thus, IOP drop during the phacoemulsification stage of our phaco + ppV also could have resulted in the postoperatively increased CVI. In addition, IOP stability during the vitrectomy might be affected when cataract surgery was done before, possibly leading to changes in CVI. However, conclusive evidence has not been found out and further investigation is needed on this subject.

Based on our study results that the CVI increased significantly in the phaco + ppV group in the first 3 months even though the CVI reduction has been reported after vitrectomy as mentioned above, it can be deduced that the effect of cataract surgery is greater than that of vitrectomy on the choroidal vascularity in the early stage of postoperative period. The exact meaning or use of the CVI change in predicting the prognosis of ocular surgery has not yet been found out. However, because it is known that the CVI increases under inflammatory conditions and IOP decrease during cataract surgery, it might be necessary to pay more attention to postoperative inflammation control and IOP during and after the surgery when cataract surgery and vitrectomy was done together.

This study had some limitations. First, some of the study subjects in the ppV group had previously received cataract surgery, which might have affected the baseline CVI. However, we tried to resolve this limitation as much as possible by excluding the eyes with history of cataract surgery in the preceding 1 year. Second, we selected the normal fellow eye as a control group for analyzing the CVI differences in ERM or FTHM. This was to minimize the interpersonal CVI difference, but an intrapersonal CVI difference could have existed and affected the study results. Also, the baseline CVI between the ppV and phaco + ppV groups were significantly different. This is one of the limitations of the study, since the difference in the baseline CVI between the two groups could have affected the CVI changes postoperatively. However, because our main purpose was to evaluate the tendency of CVI changes within each group and not to directly compare the two groups, the baseline CVI difference may be considered as a non-critical factor. Of course, further studies should be carried out considering these limitations.

In conclusion, vitrectomy significantly decreases CVI in vitreomacular diseases such as ERM and FTMH, whereas combined surgery of vitrectomy and cataract surgery significantly increases CVI in the same diseases in the early stage of the postoperative period. Although the CVI were measured lower than the baseline in the end, more thorough inflammation control and IOP may be essential after combined surgery of vitrectomy and cataract surgery. Also, further studies on the effects of these vitreomacular diseases on CVI are needed to reach an agreement.

## Methods

We retrospectively reviewed the medical records of 67 eyes with ERM and 65 eyes with FTMH from January 2019 to January 2020 at Seoul St. Mary’s Hospital in Korea. This retrospective study was conducted in accordance with the tenets of the Declaration of Helsinki, and all protocols were approved by the institutional review board of The Catholic University of Korea. The institutional review board of The Catholic University of Korea waived the need of informed consent due to the retrospective nature of the study.

The inclusion criteria were the presence of ERM or FTMH, which vitrectomy was done as a treatment. The exclusion criteria were as follows: (1) Eyes with any previous vitreomacular surgery, cataract surgery in the preceding 1 year, laser treatment, or ocular trauma, (2) eyes with an axial length of more than 26 mm measured by IOL master (Carl Zeiss Meditec, Jena, Germany) (3) eyes with refractive errors of more than ± 6 diopters (as spherical equivalent), (4) systemic diseases other than diabetes and hypertension that could affect the eye, (5) presence of other ocular diseases, including diabetic retinopathy, glaucoma, age-related macular degeneration, retinal vein occlusion, neurodegenerative disease or pachychoroidal pigment epitheliopathy in any eye, (6) media opacity disrupting image quality.

Medical and ophthalmologic history were recorded and ocular examinations, including best-corrected visual acuity (BCVA) evaluation (logarithm of the minimum angle of resolution scale; logMAR), refraction, non-contact pneumatic tonometry, axial length measure, slit-lamp biomicroscopy, and dilated fundus examination were done at the initial visit. OCT imaging was performed with a swept source (SS)-OCT device (DRI Triton, Topcon, Tokyo, Japan) using a 1050-nm wavelength light source, and a scanning speed of 100,000 A-scans/s. A 6-line radial pattern scan (1024 A-scans) centered on the fovea was obtained from each eye. OCT images were acquired at the initial visit (baseline), 1, 3 and 6 months after surgery, by a single experienced technician who was not involved in the data analysis. The distance between the outer border of the retinal pigment epithelium and the inner border of the suprachoroidal space at the foveal center was manually measured to obtain subfoveal choroidal thickness (SFCT), using digital calipers provided by the OCT software.

CVI was measured using the selected SS-OCT scan passing through the central fovea, and the scan was segmented by following the protocol described by Agrawal et al.^20^ Image binarization was done using Image J software (Version 1.51; https://imagej.nih.gov/ij/). The polygon selection tool was used to select the total choroidal area (TCA) for total macular region, and regions of interest were added to the ROI manager. The image was then converted into 8 bit, and by applying a Niblack auto-local threshold tool, separated to the luminal or vascular area (LA) and the stromal area (SA). Next, the image was converted back to an RGB (red, green, blue) image, and the LA was determined by applying the color threshold tool and added to the ROI manager. To measure the LA within the initially selected polygon, both the areas previously added in the ROI manager were selected and combined. The CVI is then measured by calculating the ratio of LA to TCA.

The surgeries were done by one experienced surgeon (Y.H.P.). 25-gauge pars plana vitrectomy (5000 cuts/minute) with the Constellation Vision System (Alcon Laboratories, Inc., Fort Worth, TX, USA) were used for every operation. Three cannulas were placed 3.5 mm from limbus. If cataract surgery was planned, phacoemulsification of lens and intraocular lens implantation into the posterior capsular bag were performed before vitrectomy. Removal of the posterior hyaloid membrane, total vitrectomy, and epiretinal membrane peeling were performed using triamcinolone acetonide, and internal limiting membrane (ILM) peeling was done using indocyanine green (ICG) (Dongindang Pharmaceutical, Seoul, Republic of Korea). If retinal tears or holes were observed, peripheral retinal photocoagulation was thoroughly performed. Filtered air or 14% octafluoropropane was applied as endotamponade as needed. The same postoperative antibiotic (levofloxacin 1.5%) and anti-inflammatory (loteprednol 0.5%) eye drops were used for all the study patients for 2 months.

We investigated the differences among the CVI measurements at baseline, 1, 3 and 6 months after surgery in ppV only group (ppV group) and in ppV combined with cataract surgery using phacoemulsification group (phaco + ppV group). As a sub-analysis, the CVI differences between normal eyes and eyes with vitreomacular diseases, which includes ERM and FTMH were investigated. This was done by selecting and analyzing 50 ERM and 51 FTMH patients whose fellow eyes were normal among the 132 study patients.

The statistical analyses were performed using the Statistical Package for the Social Sciences for Windows ver. 22.0 (SPSS Inc., Chicago, IL, USA). To analyze the differences among the CVI at baseline, 1 and 3 months after surgery, we applied repeated measures ANOVA tests and post hoc analyses. To compare the baseline and the final CVI at 6 months after surgery, we applied paired t-tests and in the process, we excluded 3 patients from phaco + ppV group and 4 patients from ppV group who did not revisit our clinic at 6 months of time. Independent t-tests were applied to compare the CVI differences between normal eyes and eyes with vitreomacular diseases. *P* values < 0.05 were considered statistically significant.
